# Age-related patterns in mandibular third molar eruption: presenting new forensic age assessment reference data from a Chinese population

**DOI:** 10.1007/s12024-024-00820-9

**Published:** 2024-05-10

**Authors:** Maximilian Timme, Jan Viktorov, Laurin Steffens, Adam Streeter, André Karch, Yu-Cheng Guo, Andreas Schmeling

**Affiliations:** 1 Institute of Legal Medicine, University Hospital Münster, Röntgenstraße 23, 48149 Münster, Germany; 2 Institute of Epidemiology and Social Medicine, University of Münster, Domagkstraße 3, 48149 Münster, Germany; 3 Department of Orthodontics, Stomatological Hospital of Xi’an Jiaotong University, 98 XiWu Road, Xi’an, 710004 Shaanxi People’s Republic of China

**Keywords:** Age estimation, Dental age, Wisdom tooth, Orthopantomogram, Chronological age

## Abstract

Valid reference data are essential for reliable forensic age assessment procedures in the living, a fact that extends to the trait of mandibular third molar eruption in dental panoramic radiographs (PAN). The objective of this study was to acquire valid reference data for a northern Chinese population. The study was guided by the criteria for reference studies in age assessment.

To this end, a study population from China comprising 917 panoramic radiographs obtained from 430 females and 487 males aged between 15.00 and 25.99 years was analysed. Of the 917 PANs, a total of 1230 mandibular third molars were evaluated.

The PANs, retrospectively evaluated, were performed for medical indication during the period from 2016 to 2021. The assessment of mandibular third molars was conducted using the staging scale presented by Olze et al. in 2012. Two independent examiners, trained in assessing PANs for forensic age estimation, evaluated the images. In instances where the two examiners diverged in their assessments these were subsequently deliberated, and a consensus stage was assigned.

The mean age increased with higher stages for both teeth and both sexes. The minimum age recorded for stage D, indicating complete tooth eruption, was 15.6 years in females and 16.1 years in males. Consequently, the completion of mandibular third molar eruption was observed in both sexes well before reaching the age of 18. In light of our results, it is evident that relying solely on the assessment of mandibular third molar eruption may not be sufficient for accurately determining the age of majority. Contrary to previous literature, this finding of a completed eruption of the mandibular third molars in northern Chinese individuals is only suitable for detecting the completion of the 16th year of life in males according to our results. However, as the results are inconsistent compared to other studies in the literature, the trait should not be used as the only decisive marker to prove this age threshold in males from northern China.

## Introduction

Forensic age assessment encompasses a wide range of approaches and methods [1–6]. It is currently an extensively researched domain within forensic sciences [7–16].

Age assessment essentially involves deducing the chronological age of an individual based on observable traits. The data collected from the individual are subsequently compared with reference data from the literature, enabling an inference about the chronological age of the individual. The relevance of reliable reference data in forensic age assessment procedures is apparent [2, 17–19].

A diverse array of trait systems, encompassing aspects of sexual maturity, skeletal development, and genetic traits, is documented in the literature for the purpose of forensic age assessment [6, 20–23]. These traits can be either developmental, such as specific stages of skeletal development and dental development, or degenerative, such as age-related genome degeneration or degenerative dental traits [24–29]. The notable strength of developmental traits resides in their primary genetic determination [30–32]. In contrast, degenerative traits are often more dependent on external influences and the transition from age-associated trait to pathological alteration is gradual. However, degenerative traits must be accessed when development is finished.

Forensic age assessment can be fundamentally categorized into two distinct applications. Firstly, in cases involving unidentified deceased individuals, where it is necessary to determine the age at the time of death, forensic age assessment can assist in elucidating the circumstances of death and in facilitating identification [33–38]. The methods applicable in these cases are broadly defined, allowing, for instance, invasive procedures as the extraction and preservation of teeth or osteological examinations of bones. Considerations related to radiation protection are not required in the case of deceased individuals.

The second major application field of forensic age assessment procedures is age assessment in living individuals [39, 40]. In instances where the age of a person is unknown or legitimate doubts exist regarding the provided age information, forensic age assessment can contribute to determining the individual’s age and providing legal certainty [39]. It is essential to note that the limitations for such procedures are considerably greater than for those on deceased individuals. For instance, considerations related to radiation protection must be taken into account [41]. Furthermore, the extraction of teeth for the purpose of forensic age assessment is precluded.

In addition to the broad categorizations of age assessment procedures in age assessment based on developmental or degenerative characteristics and its application in deceased or living individuals, another crucial aspect of forensic age estimation can be derived from the underlying aim of the process. On the one hand, forensic age assessment can be used to estimate the most probable chronological age of a person [41]. The assessment of this most probable age is possible with an acceptable margin of uncertainty. This uncertainty usually spans a few years across all methods. However, the most probable age of a person can only be estimated as long as the underlying trait is not completely developed [42].

On the other hand, the exceeding of forensically relevant age limits can be verified. The term “minimal age concept” has become established for this purpose [41]. For this approach, the minimum age that was found for the corresponding trait expression, e.g. a certain stage of development, in the relevant reference population is crucial. If several trait systems are examined, the highest minimum age found is the deciding age according to current recommendations. For expert reports in forensic age assessment, it is useful to state the minimal estimated legal age and the most probable estimated age of the individual under assessment [41].

The present study focuses on the dental developmental trait of eruption of mandibular third molars in panoramic dental radiographs (PANs). This feature represents a well-examined trait of dental development. The fundamental suitability of this trait for forensic age assessment is substantiated by strong evidence [43–48].

The trait can be employed for forensic age assessment in both living and deceased individuals, with the limitation residing in the practical execution of a PAN in the case of a deceased person.

Moreover, the trait is suitable for determining both the most probable chronological age of an individual and for surpassing specific age thresholds with the highest degree of certainty.

Various staging scales have been introduced for the assessment of mandibular third molar eruption in PAN [47, 49–51].

The aim of the study was to collect reference data for the trait of mandibular third molar eruption in PAN in a Chinese population. The research question, in particular, was whether examination of mandibular third molar eruption in PAN is suitable, following the minimal age principle, to demonstrate the attainment of 16 and 18 years of age in Chinese people.

## Material and method

The PANs used in this study were collected from the Department of Oral Radiology of the Stomatological Hospital of Xi’an Jiaotong University, Xi’an, Shaanxi, China. Importantly, all the PANs utilized in this study were originally acquired for medical purposes. The sample of PANs for this study was randomly selected for retrospective, blinded evaluation, stratified by each year of age between 15 and 25 years, which is in a line with comparable publications on the subject [47].

Age was defined in a way that the age group “15 years” includes, for example, people aged between 15.00 and 15.99 years. Leap years were taken into account for the calculation of age. The formula for calculating age was: (Date of X-ray - Date of Birth) / 365.25. For inclusion in the study, age of the participants at the time of the X-ray examination had to be known beyond doubt.

The first step was to collect the X-ray images. The selection criteria were the appropriate age and sex and the radiographic detectability of at least one mandibular third molar. The specific exclusion criteria for the individual teeth were then applied as part of the evaluations. Exclusion criteria involved inadequate image quality (e.g., artifact-related issues or misalignments in the X-ray machine resulting in image distortions), excessive retention of teeth, and pathological alterations in the teeth or jaw. The assessment of retentions adhered to established clinical criteria, such as an angle exceeding 30 degrees in the mesio-distal direction, which served as an exclusion criterion [52, 53]. Specifically, all PANs indicating pathologies such as bone fractures, cysts, carious lesions on the third molars, dental restorations on the third molars, or orthodontic appliances on the third molars were also excluded. Multiple PANs from the same individual were not permitted for inclusion.

The evaluations were performed according to the classification *by Olze et al. (2012)* [49]:

A coverage of the occlusal surface with alveolar bone.

B alveolar emerge, complete resorption of the alveolar bone over the occlusal surface.

C at least half the crown length of the second molar has been reached, the occlusal plane has not been reached.

D complete emerge in the occlusal plane.

Radiographs were examined using synedra Personal View software version 22.0.0 (synedra information technologies GmbH, Innsbruck, Austria) at appropriate workstations. The setup and ambient conditions were consistent for both examiners. During the evaluations, the software’s magnification tool and Gray-Level adjustment tool were utilized. The examiners, two board-certified dentists, possessed comprehensive familiarity with the methodology from prior studies [54]. The radiographs were independently evaluated by both examiners. Following the evaluations by both examiners, consensus was achieved through subsequent arbitration between the two examiners in cases where different stages had been determined.

Data management and statistical analyses were conducted using Stata, version 13.0 (Stata Corp LP, College Station, Texas, USA). Mean value, median, upper and lower quartile as well as the minimum and maximum age were determined for each stage.

## Results

A total of 917 PANs were included in the study, comprising 430 females and 487 males, with ages ranging from 15.00 (male) to 25.99 (male) years. The images were acquired between January 2016 and April 2021. Table 1 illustrates the composition of the study population stratified by age and biological sex.

**Table 1:** Age and sex distribution of the study population

Age	Males (*n*)	Females (*n*)	Total (*n*)
15	34	33	67
16	39	32	71
17	40	49	89
18	42	35	77
19	39	32	71
20	42	45	87
21	51	44	95
22	51	36	87
23	51	45	96
24	57	38	95
25	41	41	82
Total (n)	487	430	917

Based on the number of radiographs collected, a total of 1834 teeth could potentially have been analyzed. After strict application of the exclusion criteria, 1230 teeth (males: 630, females: 600), could finally be considered (Table 2). These 1230 teeth ultimately form the basis for the calculation of the reference data. The number of teeth examined for each third molar position in each year of age by sex ranged from *n* = 21 (tooth 48, age group 19 years, males and females) to *n* = 37 (tooth 38, age group 17 years, females) (Table 2).

**Table 2:** Number of teeth evaluated by age and sex

Age	Tooth [FDI]	Males (*n*)	Females (*n*)	Total (*n*)
15	38	30	29	59
	48	28	26	54
16	38	26	30	56
	48	27	29	56
17	38	28	37	65
	48	28	36	64
18	38	28	22	50
	48	24	24	48
19	38	22	25	47
	48	21	21	42
20	38	26	30	56
	48	27	28	55
21	38	33	28	61
	48	31	29	60
22	38	32	23	55
	48	32	25	57
23	38	33	29	62
	48	33	25	58
24	38	35	25	60
	48	34	24	58
25	38	26	29	55
	48	26	26	52
Total (n)		630	600	1230

Mean age at stage increased with stages for both sexes. For the median, this does not apply for males at tooth 38 [FDI] in the transition from stage C to D.

Among the females, the minimum age at stage D for both teeth was 15.64y, provided by the same individual (Tables 3 and 4). The minimum age at stage D for both teeth came from the same individual at 16.05y (Tables 5 and 6). Thus, the minimum ages for stage D, which correspond to a completed eruption, were well below 18 years of age for both teeth and both sexes.

**Table 3:** Descriptive measures for each stage for tooth 38 [FDI] in females. Age in years, rounded to second decimal place. SD: standard deviation. LQ: lower quartile. UQ: upper quartile

Stage	*N*	Mean	SD	Median	LQ	UQ	Min	Max
A	92	17.42	1.97	17.07	16.01	18.37	15.01	25.20
B	50	19.77	2.95	19.22	17.41	22.34	15.10	25.66
C	64	21.72	2.59	21.92	20.20	23.87	15.47	25.73
D	101	22.46	2.39	22.63	21.05	24.19	15.64	25.95

**Table 4:** Descriptive measures for each stage for tooth 48 [FDI] in females. Age in years, rounded to second decimal place. SD: standard deviation. LQ: lower quartile. UQ: upper quartile

Stage	*N*	Mean	SD	Median	LQ	UQ	Min	Max
A	93	17.47	1.98	17.13	16.07	18.29	15.01	24.01
B	32	19.47	2.42	19.45	17.24	20.75	16.06	24.71
C	71	21.61	2.78	21.86	19.37	23.86	15.23	25.73
D	97	22.48	2.38	22.52	21.05	24.47	15.64	25.95

**Table 5:** Descriptive measures for each stage for tooth 38 [FDI] in males. Age in years, rounded to second decimal place. SD: standard deviation. LQ: lower quartile. UQ: upper quartile

Stage	*N*	Mean	SD	Median	LQ	UQ	Min	Max
A	62	17.40	2.36	16.68	15.82	18.28	15.00	25.95
B	31	18.50	3.16	17.67	15.59	21.04	15.00	24.51
C	70	21.79	2.83	22.40	19.26	24.04	15.26	25.90
D	156	21.91	2.46	22.03	20.14	23.87	16.05	25.99

**Table 6:** Descriptive measures for each stage for tooth 48 [FDI] in males. Age in years, rounded to second decimal place. SD: standard deviation. LQ: lower quartile. UQ: upper quartile

Stage	*N*	Mean	SD	Median	LQ	UQ	Min	Max
A	57	17.22	2.34	16.41	15.83	17.67	15.00	24.47
B	31	18.52	2.99	18.20	15.82	20.04	15.00	25.83
C	63	21.67	2.36	21.66	19.79	23.67	16.88	25.65
D	160	22.01	2.63	22.14	20.40	24.08	16.05	25.99

The mean and median age for females in stage B and higher was above 18 years. For males, this only applied to tooth 48. For tooth 38, the mean in stage B was above 18 years, but the median was below this at 17.67 years.

The minimum age of 15.00 years for males in stages A and B for both teeth represented the lower age limit of the study sample. For females, the minimum age for all stages in both teeth were close to 15 years.

It was also noteworthy that the maximum age for all stages, including stage A, was over 24.00 years for both teeth and both sexes.

Individuals per stage ranged from 31 cases (stage B, teeth 38 and 48, males) to 160 cases (stage D, tooth 48, males). Stage D was the most frequently detected in all teeth and both sexes, largely reflecting the age distribution of the sample (Tables 3, 4, 5 and 6). Females had the greater proportion of earlier staging with stage A being detected over 90 times for both teeth (Tables 3, 4, 5 and 6).

## Discussion

The aim of generating current reference data from a northern Chinese population for eruption of mandibular third molars in PANs was achieved, and we expect that our study will contribute to a better understanding of the distribution of age within the trait and its legal relevance in the relevant population.

For northern Chinese people, few reference data are available for determining mandibular third molar eruption in PAN for forensic age assessment. In 2014, Guo et al. conducted a study on this topic [44]. They examined 1135 PANs from 506 males and 629 females aged 11–26 years. It should be emphasized that the images came from the same institution as those used for our study. Guo et al. examined X-ray images taken in 2012 and 2013 [44].

Table 7 includes the descriptive measures for stages from Guo et al. [44]. Looking at Table 7, it is evident that the values of Guo et al. for stages A and B are considerably lower than the current values of our study. Only the values for stage D, which represents a complete eruption, are around the age of 22 in both studies and are therefore quite close. It is also noteworthy that Guo et al. achieved significantly lower values for the standard deviation, except for stage D (Table 7).

**Table 7:** Comparison of the studies investigating eruption of mandibular third molars using the Olze et al. method in Asian populations and the present study. SD: standard deviation. LQ: lower quartile. UQ: upper quartile. - : No information given. Values for stage C in *italics* because the staging scale for stage C in the present study is not the same. Values rounded to one decimal place

	Guo et al. 2014 [44]	Yusof et al. 2015 [55]	Olze et al. 2008 [45]	Present study
General characteristics				
Individuals (n) | Teeth included (n) Total	1135 | -	714 | -	1300 | -	917 | 1230
Females	629 | -	373 | -	751 | -	430 | 600
Males	506 | -	341 | -	549 | -	487 | 630
Age groups [years]	11–26	14–23	14–26	15–25 (rounded up to 26.0)
Geographic origin of the study population	China (Northern China)	Malaysia	Japan	China (Northern China)
Study design	retrospective	retrospective	retrospective	retrospective
Staging scale	Olze et al. 2007 [47]	Olze et al. 2007 [47]	Olze et al. 2007 [47]	Olze et al. 2012 [49]
Results				
Intra-rater agreement [kappa]	0.93	0.92	0.88–0.96	Consensus
Inter-rater agreement [kappa]	-	0.87	-	Consensus
Descriptive measures Mean; SD | Min; Max | LQ; Median; UQ				
Stage A Sex; tooth [FDI]				
Males; 38	12.1; 1.1 | - | -	15.3; 0.9 | - | -	19.2; 3.3 | 14.6; 24.3 | 16.2; 19.2; 22.5	17.4; 2.4 | 15.0; 26.0 | 15.8; 16.7; 18.3
Males; 48	12.2; 1.1 | - | -	15.8; 0.9 | - | -	18.9; 2.9 | 14.6; 23.8 | 16.3; 19.5; 21.1	17.2; 2.3 | 15.0; 24.5 | 15.8; 16.4; 17.7
Females; 38	12.5; 1.3 | - | -	15.4; 1.2 | - | -	19.5; 2.9 | 14.2; 24.2 | 17.0; 19.0; 22.2	17.4; 2.0 | 15.0; 25.2 | 16.0; 17.1; 18.4
Females; 48	12.5; 1.3 | - | -	15.1; 0.7 | - | -	18.4; 2.8 | 14.2; 24.2 | 16.0; 18.5; 19.8	17.5; 2.0 | 15.0; 24.0 | 16.1; 17.1; 18.3
Stage B				
Males; 38	14.7; 2.2 | - | -	16.2; 2.0 | - | -	21.7; 2.2 | 18.0; 25.4 | 19.3; 21.4; 23.7	18.5; 3.1 | 15.0; 24.5 | 15.6; 17.7; 21.0
Males; 48	14.5; 1.8 | - | -	15.7; 1.1 | - | -	22.1; 2.1 | 18.0; 25.4 | 20.9; 21.8; 23.9	18.5; 3.0 | 15.0; 25.8 | 15.8; 18.2; 20.0
Females; 38	14.9; 2.1 | - | -	17.5; 2.6 | - | -	20.9; 2.2 | 15.2; 25.3 | 19.3; 20.6; 22.6	19.8; 3.0 | 15.1; 25.7 | 17.4; 19.2; 22.3
Females; 48	14.9; 2.0 | - | -	17.0; 2.2 | - | -	21.2; 2.1 | 16.5; 26.2 | 19.4; 20.8; 22.7	19.5; 2.4 | 16.1; 24.1 | 17.2; 19.5; 20.8
Stage C				
Males; 38	20.8; 3.2 | - | -	17.9; 2.0 | - | -	21.6; 2.3 | 18.1; 24.9 | 19.1; 21.8; 23.6	*21.8; 2.8 | 15.3; 25.9 | 19.3; 22.4; 24.0*
Males; 48	21.4; 2.9 | - | -	16.6; 1.1 | - | -	21.3; 2.4 | 16.8; 25.9 | 19.5; 20.9; 23.2	*21.7; 2.4 | 16.9; 25.7 | 19.8; 21.7; 23.7*
Females; 38	21.2; 3.3 | - | -	18.4; 2.5 | - | -	21.6; 2.1 | 18.1; 25.9 | 19.9; 21.6; 23.0	*21.7; 2.6 | 15.5; 25.7 | 20.2; 21.9; 23.9*
Females; 48	21.2; 3.3 | - | -	17.8; 2.5 | - | -	21.2; 1.8 | 18.1; 24.5 |19.6; 21.0; 22.7	*21.6; 2.8 | 15.2; 25.7 | 19.4; 21.9; 23.9*
Stage D				
Males; 38	22.3; 2.8 | - | -	20.8; 1.8 | - | -	22.5; 2.1 | 17.6; 26.5 | 20.7; 22.8; 24.1	21.9; 2.5 | 16.1; 26.0 | 20.1; 22.0; 23.9
Males; 48	22.2; 2.7 | - | -	20.8; 1.7 | - | -	22.5; 1.9 | 17.9; 26.6 | 20.8; 22.8; 24.0	22.0; 2.6 | 16.1; 26.0 | 20.4; 22.1; 24.1
Females; 38	22.6; 2.4 | - | -	20.5; 2.3 | - | -	22.2; 1.8 | 17.6; 26.2 | 21.0; 22.2; 23.6	22.5; 2.4 | 15.6; 26.0 | 21.1; 22.6; 24.2
Females; 48	22.6; 2.5 | - | -	20.4; 2.1 | - | -	22.2; 1.7 | 17.6; 25.8 | 21.0; 22.2; 23.5	22.5; 2.4 | 15.6; 26.0 | 21.1; 22.5; 24.5

As one conclusion of their study, Guo et al. 2014 stated that assessing the eruption of mandibular third molars in the PAN is suitable for detecting the completion of 16 years of age [44]. This was based on a binary logistic regression analysis. For females, they found a sensitivity of 100%. For males, this value was 98.9% (tooth 38) or 99.6% (tooth 48). Based on this data, it is evident that the minimum age in stage D was also below 16 years in the study by Guo et al. However, there is no specific information on this in the corresponding publication from 2014. With regard to our results, this needs to be assessed against the background of the minimum age concept [41]. Since the minimum age of females in stage D in our study was below 16 years, the result of Guo et al. for females cannot be confirmed. Assessing the eruption of mandibular third molars in the PAN cannot be used to verify the completion of 16 years of age in females. For males, the minimum age in stage D for both teeth was just over 16 years according to our results. Thus, following the minimum age concept [41], the characteristic would theoretically be suitable for proving that Chinese males had reached the age of 16. Summarizing the results of both studies, as the age in our results was only very slightly above 16 years and Guo et al. did not find 100% sensitivity in males in 2014, the completion of eruption of the mandibular third molars should not be relied upon as the exclusive marker for verifying the completion of 16 years of age in northern Chinese males. Rather, it should only be evaluated in combination with other traits.

In addition to the study by Guo et al. from 2014 in a northern Chinese population [44], there are a few other studies on assessing eruption of third molars in PANs in Asian populations. Yusof et al. conducted a study on a Malaysian population in 2015 [55]. In 2008, Olze et al. presented a study on a Japanese population [45]. Table 7 compares the studies with our present study.

In their 2015 study, Yusof et al. included a total of 714 PANs from individuals aged between 14.1 and 23.9 years [55]. Like Guo et al. [44], Yusof et al. carried out a binary logistic regression analysis and stated that stage D is particularly suitable for detecting the completion of the 18th year of life in males. Yusof et al. provided a value for a correct age prediction as a percentage of 93.6 for tooth 38 and 93.9 for tooth 48 for this age threshold. For females, these values were well below 90%. Yusof et al. did not present comparable values for the age threshold of 16, as presented by Guo et al. [44, 55]. Overall, Yusof et al. also found an accelerated eruption in females [55].

Furthermore, it is striking that the mean value in stage D of Yusof et al. is considerably lower than the values of the other studies included in Table 7.

Olze et al. conducted a study in 2008 with a total of 1300 PANs from people aged 14–26 years (Table 7) [45]. They provide comparable descriptive measures to those in our study. Olze et al. found a minimum age of over 17 years for the complete eruption of mandibular third molars (stage D) in both teeth and both sexes. The method would therefore have been suitable for proving the age of 16 years but not to prove completion of the 18th year of age. According to our results, the values for completed eruption are more than one year lower for Chinese males and two years lower for Chinese females.

Important aspects must be considered when comparing the descriptive measures of the studies in Asian populations in Table 7 to deduce any potential population-specific differences in the temporal patterns of the eruption.

These aspects include the fact that the different studies did not use the same staging scale. In the studies by Guo et al., Yusof et al. and Olze et al., a staging scale presented by Olze et al. in 2007 was used (Table 7) [47]. The scale only differs in the definition of stage C from the staging used in the present study [47, 49]. Olze et al. once noticed a shortcoming in their staging scale from 2007, so the staging scale used in the present study was then presented as a further development in 2012 [49]. The shortcoming of the 2007 staging scale concerns the definition of gingival eruption. In the 2007 scale, the criteria for assessing gingival eruption involved the penetration of at least one cusp tip of the erupting third molar through the gingival tissue [46]. Although the clinical identification of this stage presented no challenges in practical clinical settings, the radiographic evaluation of gingival eruption proved to be intricate, often rendering it unfeasible [49]. However, stages A, B and D are identical for both staging scales. This allows the reference values for stage D, which corresponds to a completed eruption in both scales, to be compared. Recently, our group demonstrated that the staging scale presented by Olze et al. in 2012 is particularly suited [54]. We were able to conclude that this staging scale should be employed for further studies in the field [54].

Other differences between the studies could explain the differing descriptive measures. Previous research has demonstrated that the age intervals of the study populations influence the mean values of the ages in the stages [17, 56]. The age range in the study by Guo et al. starts at 11 years and is therefore considerably lower than the 15 years in our study. It is obvious that this different age range alone results in lower mean values, especially for the lower stages [17, 56]. However, the age ranges between the studies by Yusof et al., Olze et al. and our study are more comparable.

In addition to the influence of different age ranges in the study populations, the influence of an uneven age distribution in the study population must also be taken into account [17]. The effect of uneven age distribution is qualitatively similar to the choice of age ranges in the study. A disproportionate number of younger individuals naturally leads to lower estimated ages for groups that include those individuals, while the greater number of older individuals increases the estimated ages [17].

Analyzing the age distribution in the study by Guo et al., it is apparent that there was a clear surplus of individuals in the 11–15 years age groups [44]. For the study by Yosuf et al., no such clear trend can be found for the age distribution in the study population, although the age groups are not represented equally here either [55]. The study by Olze et al. shows a clear overrepresentation of the 19–24 years age groups in both sexes, but particularly among females [45]. The margins of the study population, age groups 14, 15 and 26 years, are significantly underrepresented [45] in the Olze et al. study. For our study, a slight preponderance of males between the ages of 21 and 24 was found (Table 1). However, looking at the teeth examined in Table 2, the effect is not fully reflected. There is a greater underrepresentation of teeth in the 19 years age group.

The conclusion that different results between the studies can be attributed to a fundamental ethnic difference in the expression of the trait may therefore only be drawn after careful evaluation of the relevant study parameters. For studies designed to evaluate such relationships, the parameters of the studies would have to be comparable as far as possible.

When comparing the studies overall, according to our results, the assessment of mandibular third molars in PAN in females is not suitable for detecting the completion of the 16th year of life in northern Chinese individuals. With this result we could not contribute to a confirmation of the results of Guo et al. and Olze et al. for other Asian populations [44, 45].

In males, our minimum age in stage D was above 16 years, but only marginally so. However, considering the study by Guo et al. from 2014, the assessment of third molar eruption in the PAN in northern Chinese males should not be used as the decisive marker to prove the completion of 16 years of age [44]. This is in clear contrast to the results of Olze et al. from 2008 for a Japanese population [45]. When looking at the minimum ages of different studies, however, it must be noted that the lowest minimum age found across the various studies should be regarded as the “true” minimum age for the respective stage. The increase in the number of individuals examined added in the new studies increases the probability of finding the “true” minimum age.

Overall, compared to the existing literature in Asian populations, we demonstrated completion of the eruption (stage D) in both sexes at considerably younger ages [45, 47]. Future studies should therefore examine whether the postulate made by Olze et al. in 2007 that the eruption of the third molars in Asians is completed after that of Europeans and black Africans can still be assumed to be correct [47].

For our study, we chose the approach of consensual final evaluation. In existing studies on forensic age assessment, in contrast, it is common practice to determine examiner agreement and to specify corresponding coefficients [57–59] (Table 7). However, as there is never an exact agreement between the examiners in those cases, it must be assumed that “incorrect” findings are also included in the statistics and thus form the basis for the published reference values. In order to overcome this shortcoming, a consensual joint assessment was carried out in our study in cases where the two examiners had not determined the same stage. These determinations then form the basis for the reference data presented. This approach attempts to reduce the proportion of “incorrect” determinations to make the reference data more robust. However, even with this approach, it is not possible to ensure that the “true” stage was found in every case. A residual subjectivity also remains inherent in this approach.

In the study mentioned above, in which our group compared different staging scales to assess the eruption of mandibular third molars, we also examined observer agreement. The examiners were the same as in the current study.

Krippendorff coefficients of 0.904 (males) and 0.898 (females) were found for the intra-observer agreement for the Olze et al. method used in the present study. The comparable values for inter-observer agreement were 0.797 and 0.792 respectively [54]. ]. These data are within the range of comparable studies (Table 7).

For reference studies in age assessment, an equal distribution across the age groups is required [17, 39]. The required equal distribution in studies with dental traits therefore refers more to the equal distribution of teeth across the age groups. Since it is not directly possible to draw conclusions about the number of teeth examined when studying PANs and considering specific exclusion criteria, it makes sense to state the number of teeth actually examined. We have complied with this requirement and provide the relevant data in Table 2. Minor deviations observed across age cohorts within our population may be ascribed to methodological factors inherent in the study, such as the lack of a targeted pre-screening process for X-ray images prior to formal assessment. This must be mentioned as a limitation of the study. However, this approach was chosen because a targeted filling of the age groups contains the risk of bias. By specifically filling the age groups with suitable teeth, i.e. two healthy teeth each, there is a risk of eliminating the randomization and turning the sample into a biased one. Therefore, such an approach was not used in order to avoid distorting the results. The disadvantage of this approach is the resulting inevitable imbalance in the age groups.

The Chinese population is composed of many different ethnic groups [60]. The influence of these individual ethnic groups on the trait under study has not been clarified. This means that the significance of the present results is probably not representative of the entire Chinese population.

It is currently common practice in forensic age assessment studies of Chinese populations to further classify them as Northern, Southern or Western Chinese [44, 58, 61–63]. Differences between the various ethnic groups in China regarding third molar eruption should be studied in the future.

The main limitation of the present study relates to the reference sample. Prospective randomized population-based study populations would be desirable. However, prospective X-ray examinations are not possible for such studies for ethical reasons. Thus, in this field of science, retrospective use must regularly be made of existing data from medical facilities. Therefore, the study populations are not necessarily identical to the general population. This fact has already been discussed in detail in the relevant literature [64]. It is stated that the data can nevertheless be assumed to be reliable [64]. The need for strict inclusion and exclusion criteria is emphasized in the literature in order to keep the bias as low as possible [64]. For this reason, correspondingly strict criteria were applied in the present study.

## Conclusion

Contrary to older literature, in northern Chinese people, the eruption of mandibular third molars can be completed before the age of 16 in females. As the results are inconsistent with previous literature data, they should be validated by further studies. Based on our findings and existing literature, the trait should not be used as the decisive marker to prove exceeding this age threshold in males either.

In the future, reference studies on the subject should be standardized regarding the staging scale used, the age range and age distribution of the study population.

### Key points

1. The minimum age at completed eruption of the third molars in females was under 16 years.
2. The minimum age at completed eruption of the third molars in males was slightly over 16 years.
3. The trait should not be used as the decisive criterion to prove that the 16-years age limit has been reached for either sex.
4. Proof of majority is not possible with this trait in the population studied.

## Data Availability

The datasets generated during the current study are available from the corresponding author on reasonable request.

## References

[1] Simpson DJ, Chandra T. Epigenetic age prediction. Aging Cell. 2021;20:e13452. 10.1111/acel.13452 34415665 10.1111/acel.13452 PMC8441394

[2] Cummaudo M, De Angelis D, Magli F, Minà G, Merelli V, Cattaneo C. Age estimation in the living: a scoping review of population data for skeletal and dental methods. Forensic Sci Int. 2021;320:110689. 10.1016/j.forsciint.2021.110689 33561788 10.1016/j.forsciint.2021.110689

[3] Lewis JM, Senn DR. Forensic Dental Age Estimation: an overview. J Calif Dent Assoc. 2015;43:315–9. 26126347

[4] De Tobel J, Ottow C, Widek T, Klasinc I, Mörnstad H, Thevissen PW, Verstraete KL. Dental and skeletal imaging in forensic age estimation: disparities in current approaches and the Continuing search for optimization. Semin Musculoskelet Radiol. 2020;24:510–22. 10.1055/s-0040-1701495 33036039 10.1055/s-0040-1701495

[5] Roberts G, Lucas VS, Camilleri S, Jayaraman J, Kasper KA, Lewis JM. Questions of logic in Atlas methods of dental age estimation. J Forensic Leg Med. 2023;96:102505. 10.1016/j.jflm.2023.102505 37094462 10.1016/j.jflm.2023.102505

[6] Vila-Blanco N, Varas-Quintana P, Tomás I, Carreira MJ. A systematic overview of dental methods for age assessment in living individuals: from traditional to artificial intelligence-based approaches. Int J Legal Med. 2023;137:1117–46. 10.1007/s00414-023-02960-z 37055627 10.1007/s00414-023-02960-z PMC10247592

[7] Widek T, De Tobel J, Ehammer T, Genet P. Forensic age estimation in males by MRI based on the medial epiphysis of the clavicle. Int J Legal Med. 2023;137:679–89. 10.1007/s00414-022-02924-9 36534129 10.1007/s00414-022-02924-9 PMC10085911

[8] Merdietio Boedi R, Shepherd S, Oscandar F, Mânica S, Franco A. 3D segmentation of dental crown for volumetric age estimation with CBCT imaging. Int J Legal Med. 2023;137:123–30. 10.1007/s00414-022-02898-8 36197526 10.1007/s00414-022-02898-8 PMC9816244

[9] Bjørk MB, Kvaal SI, Bleka Ø, Sakinis T, Tuvnes FA, Haugland M-A, Eggesbø HB, Lauritzen PM. Prediction of Age older than 18 years in sub-adults by MRI segmentation of 1st and 2nd molars. Int J Legal Med. 2023;137:1515–26. 10.1007/s00414-023-03055-5 37402013 10.1007/s00414-023-03055-5 PMC10421773

[10] Švábová Nee Uhrová P, Beňuš R, Chovancová Nee Kondeková M, Vojtušová A, Novotný M, Thurzo A. Use of third molar eruption based on Gambier’s criteria in assessing dental age. Int J Legal Med. 2023;137:691–9. 10.1007/s00414-023-02953-y 36707450 10.1007/s00414-023-02953-y

[11] Timme M, Viktorov J, Steffens L, Streeter A, Karch A, Schmeling A. Dental age assessment in the living: a comparison of two common stage classifications for assessing radiographic visibility of the root canals in mandibular third molars. Int J Legal Med. 2023. 10.1007/s00414-023-03121-y 37952073 10.1007/s00414-023-03121-y PMC10861756

[12] Chitavishvili N, Papageorgiou I, Malich A, Hahnemann ML, Mall G, Mentzel H-J, Wittschieber D. The distal femoral epiphysis in forensic age diagnostics: studies on the evaluation of the ossification process by means of T1- and PD/T2-weighted magnetic resonance imaging. Int J Legal Med. 2023;137:427–35. 10.1007/s00414-022-02927-6 36565316 10.1007/s00414-022-02927-6 PMC9902329

[13] Gurses MS, Has B, Altinsoy HB, Suzen HS. Evaluation of distal tibial epiphysis and calcaneal epiphysis according to the Vieth method in 3.0 T magnetic resonance images: a pilot study. Int J Legal Med. 2023;137:1181–91. 10.1007/s00414-023-03010-4 37145316 10.1007/s00414-023-03010-4

[14] Qiu L, Liu A, Dai X, Liu G, Peng Z, Zhan M, Liu J, Gui Y, Zhu H, Chen H, Deng Z, Fan F. Machine learning and deep learning enabled age estimation on medial clavicle CT images. Int J Legal Med. 2023. 10.1007/s00414-023-03115-w 37940721 10.1007/s00414-023-03115-w

[15] Angelakopoulos N, De Luca S, Oliveira-Santos I, Ribeiro ILA, Bianchi I, Balla SB, Kis HC, Jiménez LG, Zolotenkova G, Yusof MYPM, Selmanagić AH, Pandey H, Pereira PC, da Nóbrega JBM, Kalani H, Mieke SM, Kumagai A, Gulsahi A, Zelić K, Marinković N, Kelmendi J, Galić I, Vázquez IS, Spinas E, Velezmoro-Montes YW, Moukarzel M, Toledo JP, El-Bakary AAE-S, Cameriere R. Third molar maturity index (I3M) assessment according to different geographical zones: a large multi-ethnic study sample. Int J Legal Med. 2023;137:403–25. 10.1007/s00414-022-02930-x 36520207 10.1007/s00414-022-02930-x

[16] Wang C, Tian Z, Wen D, Qu W, Xu R, Liu Y, Jia H, Tang X, Li J, Zha L, Liu Y. Preliminary study on genetic factors related to Demirjian’s tooth age estimation method based on genome-wide association analysis. Int J Legal Med. 2023;137:1161–79. 10.1007/s00414-023-03008-y 37133749 10.1007/s00414-023-03008-y

[17] Gelbrich B, Lessig R, Lehmann M, Dannhauer K-H, Gelbrich G. Altersselektion in Referenzstichproben: Auswirkung auf die forensische Altersschätzung. Rechtsmedizin. 2010;20:459–63. 10.1007/s00194-010-0703-3

[18] Sgheiza V, Liversidge HM. The effect of reference sample composition and size on dental age interval estimates. Am J Biol Anthropol. 2023;182:82–92. 10.1002/ajpa.24790 37294283 10.1002/ajpa.24790

[19] Sgheiza V, Liversidge H. Reference and target sample age distribution impacts between model types in dental developmental age estimation. Int J Legal Med. 2023;137:383–93. 10.1007/s00414-022-02925-8 36495334 10.1007/s00414-022-02925-8

[20] Malina RM, Rogol AD, Cumming SP, Coelho e Silva MJ, Figueiredo AJ. Biological maturation of youth athletes: assessment and implications. Br J Sports Med. 2015;49:852–9. 10.1136/bjsports-2015-094623 26084525 10.1136/bjsports-2015-094623

[21] Rai V, Saha S, Yadav G, Tripathi AM, Grover K. Dental and skeletal maturity- a biological indicator of chronologic age. J Clin Diagn Res. 2014;8:ZC60–64. 10.7860/JCDR/2014/10079.4862 25386525 10.7860/JCDR/2014/10079.4862 PMC4225977

[22] Carlsen L, Holländer O, Danzer MF, Vennemann M, Augustin C. DNA methylation-based age estimation for adults and minors: considering sex-specific differences and non-linear correlations. Int J Legal Med. 2023;137:635–43. 10.1007/s00414-023-02967-6 36811674 10.1007/s00414-023-02967-6 PMC10085938

[23] Bell CG, Lowe R, Adams PD, Baccarelli AA, Beck S, Bell JT, Christensen BC, Gladyshev VN, Heijmans BT, Horvath S, Ideker T, Issa J-PJ, Kelsey KT, Marioni RE, Reik W, Relton CL, Schalkwyk LC, Teschendorff AE, Wagner W, Zhang K, Rakyan VK. DNA methylation aging clocks: challenges and recommendations. Genome Biol. 2019;20:249. 10.1186/s13059-019-1824-y 31767039 10.1186/s13059-019-1824-y PMC6876109

[24] Fang C, Zhou P, Li R, Guo J, Qiu H, Zhang J, Li M, Yu C, Meng D, Xu X, Liu X, Guan D, Yan J. Development of a novel forensic age estimation strategy for aged blood samples by combining piRNA and miRNA markers. Int J Legal Med. 2023;137:1327–35. 10.1007/s00414-023-03028-8 37264192 10.1007/s00414-023-03028-8

[25] Wang ZW, Xu QN, Li CT, Liu XL. Age Estimation based on DNA methylation and its application prospects in Forensic Medicine. Fa Yi Xue Za Zhi. 2023;39:72–82. 10.12116/j.issn.1004-5619.2021.510604 37038859 10.12116/j.issn.1004-5619.2021.510604

[26] Márquez-Ruiz AB, González-Herrera L, Luna J, de Valenzuela D A. DNA methylation levels and telomere length in human teeth: usefulness for age estimation. Int J Legal Med. 2020;134:451–9. 10.1007/s00414-019-02242-7 31897670 10.1007/s00414-019-02242-7

[27] Koh KK, Tan JS, Nambiar P, Ibrahim N, Mutalik S, Khan Asif M. Age estimation from structural changes of teeth and buccal alveolar bone level. J Forensic Leg Med. 2017;48:15–21. 10.1016/j.jflm.2017.03.004 28407514 10.1016/j.jflm.2017.03.004

[28] Shrigiriwar M, Jadhav V. Age estimation from physiological changes of teeth by Gustafson’s method. Med Sci Law. 2013;53:67–71. 10.1258/msl.2012.011119 23275430 10.1258/msl.2012.011119

[29] Singh N, Grover N, Puri N, Singh S, Arora S. Age estimation from physiological changes of teeth: a reliable age marker? J Forensic Dent Sci. 2014;6:113–21. 10.4103/0975-1475.132541 25125919 10.4103/0975-1475.132541 PMC4130013

[30] Kreiborg S, Jensen BL. Tooth formation and eruption - lessons learnt from cleidocranial dysplasia. Eur J Oral Sci 126 Suppl. 2018;172–80. 10.1111/eos.12418 10.1111/eos.12418 30178560

[31] Wise GE, King GJ. Mechanisms of tooth eruption and orthodontic tooth movement. J Dent Res. 2008;87:414–34. 10.1177/154405910808700509 18434571 10.1177/154405910808700509 PMC2387248

[32] Wagner EF, Karsenty G. Genetic control of skeletal development. Curr Opin Genet Dev. 2001;11:527–32. 10.1016/s0959-437x(00)00228-8 11532394 10.1016/s0959-437x(00)00228-8

[33] Savall F, Hérin F, Peyron PA, Rougé D, Baccino E, Saint-Martin P, Telmon N. Age estimation at death using pubic bone analysis of a virtual reference sample. Int J Legal Med. 2018;132:609–15. 10.1007/s00414-017-1656-9 28770383 10.1007/s00414-017-1656-9

[34] Jooste N, Pretorius S, Steyn M. Performance of three mathematical models for estimating age-at-death from multiple indicators of the adult skeleton. Int J Legal Med. 2022;136:739–51. 10.1007/s00414-021-02727-4 34767061 10.1007/s00414-021-02727-4

[35] Oliveira-Santos I, Gouveia M, Cunha E, Gonçalves D. The circles of life: age at death estimation in burnt teeth through tooth cementum annulations. Int J Legal Med. 2017;131:527–36. 10.1007/s00414-016-1432-2 27590012 10.1007/s00414-016-1432-2

[36] Pedrosa M, Curate F, Batista de Carvalho LAE, Marques MPM, Ferreira MT. Beyond metrics and morphology: the potential of FTIR-ATR and chemometrics to estimate age-at-death in human bone. Int J Legal Med. 2020;134:1905–14. 10.1007/s00414-020-02310-3 32385593 10.1007/s00414-020-02310-3

[37] Jooste N, Steyn M. The first rib as a method of adult age-at-death estimation in a modern South African sample. Int J Legal Med. 2023;137:743–52. 10.1007/s00414-023-02978-3 36929197 10.1007/s00414-023-02978-3 PMC10085915

[38] Viciano J, Icaro I, Tanga C, Tripodi D. Influence of light conditions (colour temperature and illuminance) on the evaluation of root translucency for the application of Lamendin’s age-at-death estimation technique. Int J Legal Med. 2023;137:131–44. 10.1007/s00414-022-02902-1 36261608 10.1007/s00414-022-02902-1 PMC9816196

[39] Schmeling A, Grundmann C, Fuhrmann A, Kaatsch H-J, Knell B, Ramsthaler F, Reisinger W, Riepert T, Ritz-Timme S, Rösing FW, Rötzscher K, Geserick G. Criteria for age estimation in living individuals. Int J Legal Med. 2008;122:457–60. 10.1007/s00414-008-0254-2 18548266 10.1007/s00414-008-0254-2

[40] Black SM, Aggrawal A, Payne-James J. Age estimation in the living: the practitioners guide. Chichester, West Sussex, UK; Hoboken, NJ: Wiley-Blackwell; 2010.

[41] Schmeling A, Dettmeyer R, Rudolf E, Vieth V, Geserick G. Forensic age estimation: methods, certainty, and the Law. Deutsches Ärzteblatt International. 2016. 10.3238/arztebl.2016.0044 26883413 10.3238/arztebl.2016.0044 PMC4760148

[42] Hagen M, Schmidt S, Schulz R, Vieth V, Ottow C, Olze A, Pfeiffer H, Schmeling A. Forensic age assessment of living adolescents and young adults at the Institute of Legal Medicine, Münster, from 2009 to 2018. Int J Legal Med. 2020;134:745–51. 10.1007/s00414-019-02239-2 31907616 10.1007/s00414-019-02239-2

[43] Olze A, Peschke C, Schulz R, Schmeling A. Studies of the chronological course of wisdom tooth eruption in a German population. J Forensic Leg Med. 2008;15:426–9. 10.1016/j.jflm.2008.02.008 18761308 10.1016/j.jflm.2008.02.008

[44] Guo YC, Yan C, Lin X, Zhou H, Pan F, Wei L, Tang Z, Liang F, Chen T. Studies of the chronological course of third molars eruption in a northern Chinese population. Arch Oral Biol. 2014;59:906–11. 10.1016/j.archoralbio.2014.05.018 24907520 10.1016/j.archoralbio.2014.05.018

[45] Olze A, Ishikawa T, Zhu BL, Schulz R, Heinecke A, Maeda H, Schmeling A. Studies of the chronological course of wisdom tooth eruption in a Japanese population. Forensic Sci Int. 2008;174:203–6. 10.1016/j.forsciint.2007.04.218 17548179 10.1016/j.forsciint.2007.04.218

[46] Olze A, van Niekerk P, Schulz R, Schmeling A. Studies of the chronological course of wisdom tooth eruption in a black African population. J Forensic Sci. 2007;52:1161–3. 10.1111/j.1556-4029.2007.00534.x 17767660 10.1111/j.1556-4029.2007.00534.x

[47] Olze A, van Niekerk P, Ishikawa T, Zhu BL, Schulz R, Maeda H, Schmeling A. Comparative study on the effect of ethnicity on wisdom tooth eruption. Int J Legal Med. 2007;121:445–8. 10.1007/s00414-007-0171-9 17453230 10.1007/s00414-007-0171-9

[48] Kutesa AM, Rwenyonyi CM, Mwesigwa CL, Muhammad M, Nabaggala GS, Kalyango J. Dental age estimation using radiographic assessment of third molar eruption among 10-20-year-old Ugandan population. J Forensic Dent Sci. 2019;11:16–21. 10.4103/jfo.jfds_34_19 31680751 10.4103/jfo.jfds_34_19 PMC6822306

[49] Olze A, Peschke C, Schulz R, Schmeling A. [Application of a modified stage classification in evaluating wisdom tooth eruption in a German population]. Arch Kriminol. 2012;229:145–53. 22834358

[50] Gambier A, Rérolle C, Faisant M, Lemarchand J, Paré A, Saint-Martin P. Contribution of third molar eruption to the estimation of the forensic age of living individuals. Int J Legal Med. 2019;133:625–32. 10.1007/s00414-018-01991-1 30635722 10.1007/s00414-018-01991-1

[51] Willmot SE, Hector MP, Liversidge HM. Accuracy of estimating age from eruption levels of mandibular teeth. DAJ. 2018;26:56–62. 10.26575/daj.v26i3.52

[52] Archer WH. (1955) Die Chirurgie des Mundes und der Zähne. Medica. Stuttgart. Germany.

[53] Wolf H, Haunfelder D. (1960) Zahnärztliche Mundchirurgie für Studierende der Zahnheilkunde. Berlinische Verlagsanstalt. Berlin. Germany.

[54] Timme M, Viktorov J, Steffens L, Streeter A, Karch A, Schmeling A. Third molar eruption in orthopantomograms as a feature for forensic age assessment-a comparison study of different classification systems. Int J Legal Med. 2023;137:765–72. 10.1007/s00414-023-02982-7 36884067 10.1007/s00414-023-02982-7 PMC10085935

[55] Yusof MYPM, Cauwels R, Martens L. Stages in third molar development and eruption to estimate the 18-year threshold malay juvenile. Arch Oral Biol. 2015;60:1571–6. 10.1016/j.archoralbio.2015.07.017 26276268 10.1016/j.archoralbio.2015.07.017

[56] Knell B, Ruhstaller P, Prieels F, Schmeling A. Dental age diagnostics by means of radiographical evaluation of the growth stages of lower wisdom teeth. Int J Legal Med. 2009;123:465–9. 10.1007/s00414-009-0330-2 19241087 10.1007/s00414-009-0330-2

[57] Jayaraman J, Wong HM, King NM, Roberts GJ. Development of a Reference Data Set (RDS) for dental age estimation (DAE) and testing of this with a separate Validation Set (VS) in a southern Chinese population. J Forensic Leg Med. 2016;43:26–33. 10.1016/j.jflm.2016.07.007 27441983 10.1016/j.jflm.2016.07.007

[58] Zeng DL, Wu ZL, Cui MY. Chronological age estimation of third molar mineralization of Han in southern China. Int J Legal Med. 2010;124:119–23. 10.1007/s00414-009-0379-y 19908057 10.1007/s00414-009-0379-y

[59] Sisman Y, Uysal T, Yagmur F, Ramoglu SI. Third-molar development in relation to chronologic age in Turkish children and young adults. Angle Orthod. 2007;77:1040–5. 10.2319/101906-430.1 18004924 10.2319/101906-430.1

[60] Zhang S, Lo ECM, Liu J, Chu CH. A review of the dental caries status of ethnic minority children in China. J Immigr Minor Health. 2015;17:285–97. 10.1007/s10903-013-9916-3 24057753 10.1007/s10903-013-9916-3

[61] Si X, Chu G, Olze A, Schmidt S, Schulz R, Chen T, Pfeiffer H, Guo Y, Schmeling A. Age assessment in the living using modified Gustafson’s criteria in a northern Chinese population. Int J Legal Med. 2019. 10.1007/s00414-019-02024-1 30790037 10.1007/s00414-019-02024-1

[62] Zhang ZY, Yan CX, Min QM, Li SQ, Yang JS, Guo YC, Jin WF, Li LJ, Xing PF, Li J. Age estimation using pulp/enamel volume ratio of impacted mandibular third molars measured on CBCT images in a northern Chinese population. Int J Legal Med. 2019;133:1925–33. 10.1007/s00414-019-02112-2 31273446 10.1007/s00414-019-02112-2

[63] Zhang K, Dong X, Chen X, Li Y, Deng Z. Forensic age estimation through evaluation of the apophyseal ossification of the iliac crest in western Chinese. Forensic Sci Int. 2015;252:e1921–5. 10.1016/j.forsciint.2015.04.032 10.1016/j.forsciint.2015.04.032 25982819

[64] Roberts G, Lucas VS. (2021) Is the Demirjian, Goldstein, and Tanner Method of Dental Age Estimation obsolete? A critical review and re-assessment. Insights Anthropol 5:. 10.36959/763/519

